# Myofascial rotation technique for open fetal myelomeningocele repair

**DOI:** 10.3171/2026.1.FOCVID25235

**Published:** 2026-04-01

**Authors:** Charles E. Reilly, Mauro H. Schenone, Edward S. Ahn

**Affiliations:** Departments of 1 Neurologic Surgery and; 2 Obstetrics and Gynecology, Mayo Clinic, Rochester, Minnesota

**Keywords:** myelomeningocele, fetal, surgery, myofascial, closure

## Abstract

Fetal repair of myelomeningocele remains an effective intervention to decrease hindbrain herniation and reduce the need for postnatal CSF shunting. Effectiveness depends on a watertight closure; however, there are challenges to achieving such. Often, a separate, watertight dural closure is difficult due to the thinness and fragility of the dural membrane. This video demonstrates the approach utilizing a previously described myofascial rotation of the bilateral paraspinal muscles to construct a robust closure even without the need for a separate dural closure or patch reinforcement. This method has been associated with improved primary closure rates and fewer postnatal procedures.

The video can be found here: https://stream.cadmore.media/r10.3171/2026.1.FOCVID25235

## Transcript

### 0:30 Introduction.

Fetal repair of myelomeningocele is associated with significantly improved outcomes when compared with postnatal repair.^1^ If left untreated, myelomeningocele can cause hindbrain herniation and obstructive hydrocephalus.^2^ Here, we demonstrate the use of a previously described myofascial rotation technique for the repair of myelomeningocele during open fetal surgery.^3^

### 0:54 Patient Characteristics.

Our patient is a 35-year-old female at 25 weeks and 4 days gestation who previously delivered three children vaginally. On fetal MRI at 24 weeks we can see the lumbosacral defect at L5, as well as evidence of hindbrain herniation. There was no ventriculomegaly detected.

### 1:14 Dissection of Neural Placode.

With ultrasound guidance the myelomeningocele defect is localized and a hysterotomy is made over the top of the defect. After adjustment of the fetus into position, we see the exposed neural placode. The first step is incising the cystic membrane around the placode circumferentially. This is done until the placode is free and falls into the spinal canal.

### 1:55 Attempted Dural Closure.

Standard repair includes suturing the dura closed in a watertight fashion.^4^ We identified a thin membrane surrounding the placode that resembled the dura. We freed this membrane and attempted to approximate the edges together over top of the placode. However, the membrane was very thin and insufficient for a watertight closure.

### 2:19 Myofascial Flap Dissection.

Therefore, we decided to rotate myofascial flaps over the defect as described in Flanders et al.,^3^ which reduces the need for patch reinforcement or postnatal CSF diversion.^5^ This was completed by mobilizing the skin overlaying the paraspinal muscles and using monopolar cautery to incise the fascia and underlying muscle. This allowed us to mobilize the muscle medially. A similar incision into the fascia was made on the other side of the defect. We freed the muscle until we felt that both sides could be approximated over the defect.

### 3:40 Myofascial Flap Closure.

Interrupted sutures were used to bring the myofascial flaps together over the midline. This allowed for complete covering of the spinal defect and a watertight closure. In the senior author’s experience, myofascial rotation is consistently utilized in situations where primary dural closure is not possible. In addition, it can be applied as an adjunct to reinforce the repair in the minority of cases where a separate dural layer can be closed.

### 4:54 Dermal Closure.

After the myofascial flap was closed, we proceeded to close the skin. We mobilized the skin widely from the underlying muscular fascia. We then were able to close the skin edges together with a running barbed suture. The size of the repair was 4 cm.

### 6:00 Postoperative Course.

Follow-up postoperative fetal MRI showed complete tissue covering the previously seen myelomeningocele defect at 31 weeks gestation. MRI also demonstrated reversal of the previously seen hindbrain herniation, showing ample fluid in the cisterna magna. The baby was born at 36 weeks 5 days gestation by scheduled cesarean section without incident. The skin was completely intact without defect, and the infant did not require postnatal shunting.

## Acknowledgments

We would like to acknowledge the anesthesia team, the surgical assistants, and the videographers for their roles in the creation of this project.

## Disclosures

The authors report no conflict of interest concerning the materials or methods used in this study or the findings specified in this publication.

## Author Contributions

Primary surgeon: Ahn. Assistant surgeon: Schenone. Editing and drafting the video and abstract: Ahn, Reilly. Critically revising the work: all authors. Reviewed submitted version of the work: Ahn, Reilly. Approved the final version of the work on behalf of all authors: Ahn.

## Supplemental Information

### Patient Informed Consent

The necessary patient informed consent was obtained in this study.
